# Post-marketing safety profile of palivizumab: a 20-year pharmacovigilance study using the FDA Adverse Event Reporting System (FAERS)

**DOI:** 10.1007/s00210-026-05485-5

**Published:** 2026-06-04

**Authors:** Qing Yan, Yuke Zhong

**Affiliations:** 1 Jincheng Center for Adverse Drug Reaction Monitoring, Jincheng, 048200 Shanxi China; 2 Longgang Central Hospital of Shenzhen, Shenzhen, 518116 China

**Keywords:** Palivizumab, Pharmacovigilance, FAERS, Adverse events, Real-world evidence, Pediatric safety

## Abstract

Palivizumab is an FDA-approved monoclonal antibody for preventing severe respiratory syncytial virus (RSV) infections in high-risk children. However, its real-world safety profile, particularly beyond clinical trial populations, remains incompletely characterized. This study aimed to systematically analyze adverse events (AEs) associated with palivizumab using two decades of data from the FDA Adverse Event Reporting System (FAERS) to identify known, novel, and rare safety signals. This study aimed to characterize the post-marketing safety profile of palivizumab and identify AE signals using 20 years of FDA Adverse Event Reporting System (FAERS) data. A retrospective pharmacovigilance study was conducted using FAERS data from Q1 2004 to Q2 2024. Duplicate reports were removed, and palivizumab-related AE reports (primary suspected drug) were extracted. AEs were standardized via MedDRA v27.0 and categorized by system organ classes (SOCs) and preferred terms (PTs). Disproportionality analyses, including reporting odds ratio (ROR), proportional reporting ratio (PRR), multi-item gamma Poisson shrinker (MGPS), and Bayesian Confidence Propagation Neural Network (BCPNN), were used to detect signals, with predefined criteria for signal validation. Among 21,558,936 total FAERS reports, 14,120 (0.065%) palivizumab-related AE cases were included, predominantly in infants aged 28 days–23 months (45.92% of all cases); 51.74% of cases with known sex were male. The most frequent serious outcome was hospitalization (60.98%), followed by death (12.12%). Three system organ class–level positive signals were identified, including two novel signals (Infections and infestations; Congenital, familial and genetic disorders). The strongest preferred term–level positive signals were RSV-related events, with novel signals including infantile spitting up, post-tussive vomiting, oligodipsia, and Kawasaki’s disease. This 20-year real-world study confirms known AEs of palivizumab and identifies novel signals, particularly in infection-related and congenital disorder categories. These findings do not establish causality but highlight the need for prospective studies to validate associations, guiding safer clinical use in high-risk pediatric populations.

**Supplementary Information:**

The online version contains supplementary material available at 10.1007/s00210-026-05485-5.

## Introduction

Respiratory syncytial virus (RSV), a single-stranded, negative-sense RNA virus of the Paramyxoviridae family, is the leading global cause of acute lower respiratory tract infections (ALRTIs) in children under 5 years of age (Alenzi et al. 2024). The burden of RSV disease is disproportionately concentrated in infants and toddlers under 2 years of age, with the global incidence of RSV-associated ALRTIs estimated at 0.52% in newborns and up to 2.6% in infants within the first year of life (Cai et al. 2020; Caserta et al. 2023). The pathogenesis of RSV infection involves complex interactions between the virus, host immune responses, and risk factors (Cai et al. 2020; Caserta et al. 2023). Notably, primary RSV infection does not confer lifelong sterilizing immunity, leaving children susceptible to recurrent episodes of infection throughout early childhood (Chavez-Bueno et al. 2006).

The clinical spectrum of RSV infection is highly heterogeneous, ranging from mild upper respiratory tract symptoms (including rhinorrhea, nasal congestion, and cough) to severe, life-threatening lower respiratory tract disease such as bronchiolitis and pneumonia (Chavez-Bueno et al. 2006). Common clinical manifestations of severe infection include persistent cough, tachypnea, oral frothing, and expiratory wheezing. While the majority of immunocompetent children recover from acute RSV infection without long-term sequelae, multiple epidemiological studies have demonstrated that infants with severe RSV infection have a fourfold higher risk of developing recurrent wheezing and asthma in later childhood compared to healthy, age-matched controls (Dubrall et al. 2021). Premature infants (born at < 32 weeks’ gestation), children with hemodynamically significant congenital heart disease, chronic lung disease of prematurity, Down syndrome, or primary/secondary immunodeficiencies are at markedly elevated risk of severe RSV infection, hospitalization, and adverse clinical outcomes (Dubrall et al. 2021; Feltes et al. 2003; Fenton et al. 2004; Shi et al. 2015). Despite decades of research, there remains no globally approved curative antiviral therapy for established RSV infection, with clinical management limited to supportive care and targeted prophylactic interventions to reduce disease risk in vulnerable populations (Giunchi et al. 2023).

Palivizumab is a recombinant humanized monoclonal antibody (IgG1) directed against antigenic site II of the RSV fusion (F) protein, and was the first biological product approved by the US FDA for the prevention of severe RSV infection in high-risk pediatric patients (Giunchi et al. 2023). The mechanism of action of palivizumab involves neutralization of extracellular RSV virions and inhibition of viral fusion to the plasma membrane of respiratory epithelial cells, thereby blocking viral replication and spread within the lower respiratory tract (Giunchi et al. 2023). In 2023, the American Academy of Pediatrics (AAP) updated its clinical practice guidelines, recommending palivizumab prophylaxis for infants born at < 32 weeks’ gestation, as well as children under 2 years of age with hemodynamically significant congenital heart disease, chronic lung disease, or other conditions associated with increased RSV-related morbidity (Caserta et al. 2023). The recommended prophylactic regimen consists of monthly intramuscular injections of palivizumab at a dose of 15 mg/kg, initiated prior to the onset of the local RSV season, for a maximum of 5 doses per season (Groothuis 2003). Multiple clinical trials and real-world cohort studies have demonstrated that palivizumab prophylaxis significantly reduces the risk of RSV-related hospitalization, lower respiratory tract infection, and recurrent wheezing days in high-risk pediatric populations (Caserta et al. 2023).

In pre-approval clinical trials, palivizumab has generally been shown to be safe and well-tolerated, with a low overall incidence of treatment-emergent adverse events (AEs) ranging from 1 to 12% across study populations (Giunchi et al. 2023; Group 1998; Heilman 1990; Johnson et al. 1997; Mazur et al. 2023). The landmark IMpact-RSV trial, a randomized, double-blind, placebo-controlled phase III trial that established the efficacy of palivizumab, reported no significant difference in the overall incidence of AEs between the palivizumab and placebo groups (11% vs. 10%, respectively) (Group 1998; Heilman 1990; Johnson et al. 1997). The most commonly reported AEs in clinical trials included fever (2.8% palivizumab vs. 3% placebo), nervousness (2.5% vs. 2.6%), injection site reactions (including erythema, pain, swelling, and bruising; 2.7% vs. 1.8%), and mild to moderate elevations in aspartate aminotransferase (AST): 3.6% vs. 1.6%; alanine aminotransferase (ALT): 2.3% vs. 2% (Group 1998; Heilman 1990; Johnson et al. 1997).

However, the existing safety data for palivizumab are predominantly derived from pre-approval clinical trials, which have inherent limitations that restrict their generalizability to real-world clinical practice. Clinical trials typically enroll highly selected patient populations with strict inclusion and exclusion criteria, often excluding the most severely ill infants, those with multiple complex comorbidities, and patients receiving off-label prophylaxis (Lacaze‐Masmonteil et al. 2002). Additionally, clinical trials have relatively short follow-up periods, typically limited to a single RSV season, and are underpowered to detect rare, serious, or delayed-onset AEs that may only emerge in large, diverse populations over extended post-marketing use (Lacaze‐Masmonteil et al. 2002). These gaps in the safety evidence base highlight the critical need for large-scale, long-term post-marketing pharmacovigilance studies to characterize the real-world safety profile of palivizumab.

Spontaneous reporting systems (SRS) such as the FDA Adverse Event Reporting System (FAERS) are a cornerstone of global post-marketing drug safety surveillance. FAERS aggregates spontaneous AE reports from healthcare providers, patients, caregivers, and pharmaceutical manufacturers worldwide, providing a large, real-world data source for the detection of novel, rare, or unexpected drug safety signals that may not have been identified in pre-approval clinical trials (Lacaze‐Masmonteil et al. 2002). While spontaneous reporting data have inherent limitations (including underreporting, lack of a control population, and potential confounding by indication and comorbidities), disproportionality analysis of SRS data remains the most widely used and validated method for hypothesis generation in post-marketing pharmacovigilance (Liu et al. 2019).

To date, few large-scale, long-term pharmacovigilance studies have comprehensively evaluated the post-marketing safety profile of palivizumab using FAERS data. Therefore, the present study was designed to address this evidence gap by analyzing 20 years of FAERS data (2004 Q1 to 2024 Q2) to (1) describe the epidemiological and clinical characteristics of palivizumab-associated AE reports; (2) systematically identify and validate known, novel, and positive signals for palivizumab at both the system organ class (SOC) and preferred term (PT) levels using multiple established disproportionality analysis methods; and (3) provide real-world evidence to guide clinical prescribing and post-marketing safety monitoring of palivizumab in high-risk pediatric populations.

## Aim

This study aimed to comprehensively characterize the post-marketing safety profile of palivizumab, identify known, novel, and positive signals, and describe the epidemiological and clinical features of palivizumab-associated AEs using 20 years of data from the FDA Adverse Event Reporting System (FAERS).

## Methods

### Study design and data source

This was a retrospective, observational pharmacovigilance study using de-identified, publicly available data from the FDA Adverse Event Reporting System (FAERS). The study protocol conformed to the ethical guidelines for retrospective database research, with no requirement for institutional review board approval, as all data are de-identified and publicly accessible. This study adhered to the FDA’s FAERS data usage guidelines, and no attempt was made to identify individual patients. Clinical trial number: not applicable.

**Fig. 1:**
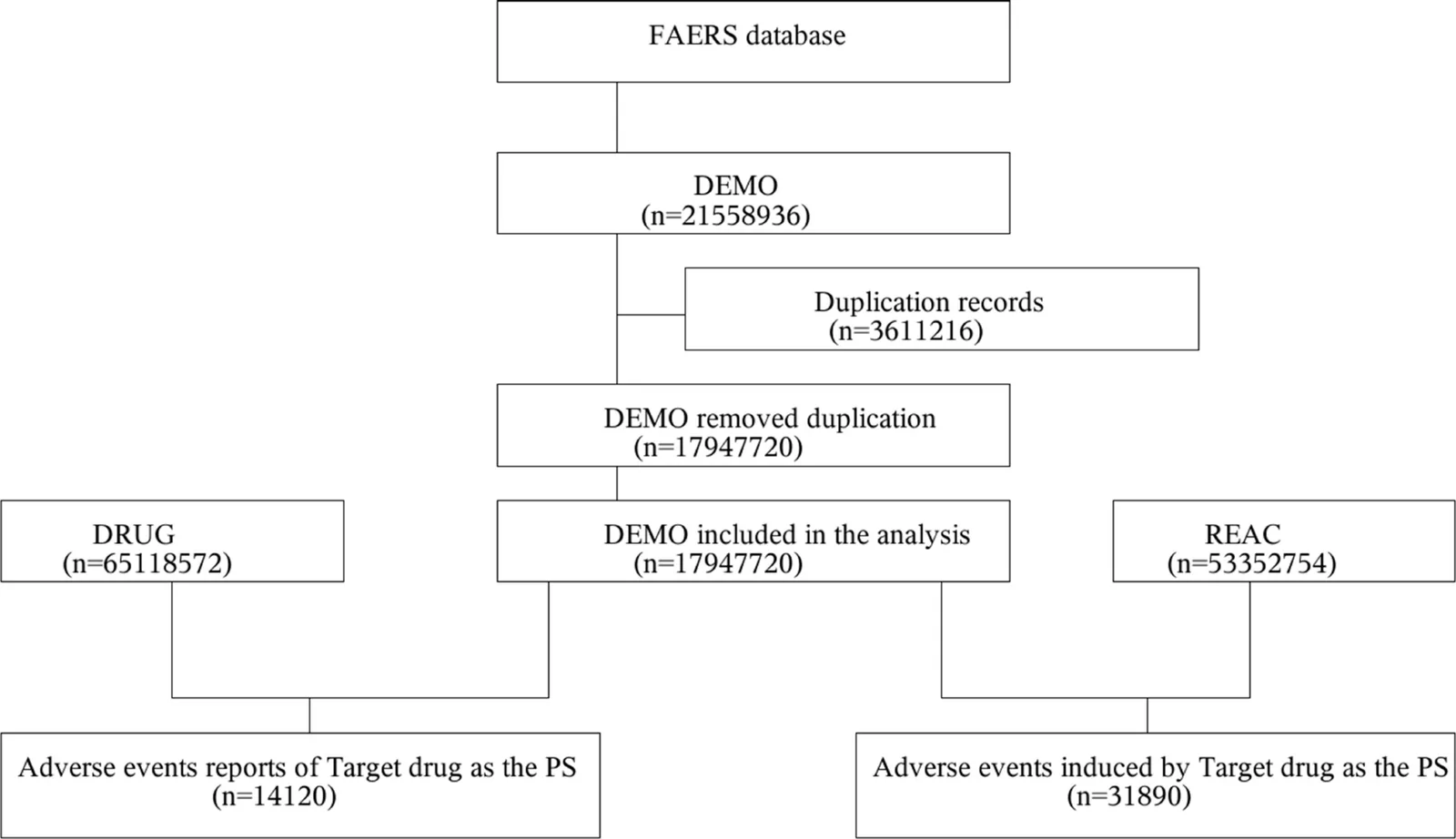
The data analysis flow chart

FAERS is a spontaneous reporting database maintained by the US FDA for the post-marketing surveillance of drug and biologic safety. The database is structured into seven quarterly data files: Demographic and Administrative (DEMO), Drug (DRUG), Reaction (REAC), Outcome (OUTC), Indication (INDI), Therapy (THER), and Source (SRSE). For this study, we extracted all quarterly FAERS data files from the first quarter (Q1) of 2004 through the second quarter (Q2) of 2024, covering a 20-year study period. The full data processing workflow is summarized in Fig. 1.

### Data extraction and preprocessing

Raw FAERS data files were imported into a MySQL 8.0 relational database management system (Oracle Corporation, Redwood Shores, CA, USA) for structured data cleaning and preprocessing. First, duplicate AE reports were identified and removed using a validated deduplication algorithm, which retained the highest-level primary report based on unique case identifiers, as recommended by the FDA for FAERS data analysis (Lacaze‐Masmonteil et al. 2002).

Next, we performed a targeted search to identify all AE reports where palivizumab (generic name) or its brand name Synagis was listed as the primary suspected drug. The primary suspected drug was defined as the drug most strongly implicated in the AE by the reporter, as specified in the DRUG file of the FAERS database. For each eligible case, we extracted the following key variables: unique safety report number, patient demographic characteristics (age, gender, weight), suspected and concomitant medications, reported indications for palivizumab use, comorbidities, AE descriptions, event outcomes, reporter type, and geographic region of the report.

### Standardization of adverse events

All reported AEs were coded and standardized using the Medical Dictionary for Regulatory Activities (MedDRA) version 27.0, the international standard terminology for drug safety reporting. AEs were categorized hierarchically at two levels: the system organ class (SOC) level (the highest level of grouping, based on anatomical or physiological systems) and the preferred term (PT) level (a single, standardized descriptor for a distinct clinical event) (Liu et al. 2023). For each AE, we mapped the reported event term to the corresponding SOC and PT code, ensuring consistency and comparability of the data across the study period.

### Statistical analysis and safety signal detection

Descriptive statistics were used to characterize the baseline demographic and clinical features of the study population, as well as the distribution of palivizumab-associated AE reports. For descriptive analyses, missing demographic data were not imputed; percentages were calculated based on the number of non‑missing cases. Categorical variables were presented as frequencies and percentages (*n*, %), while continuous variables (age, weight, time to AE onset) were presented as mean ± standard deviation (SD) and median with interquartile range (IQR, Q1–Q3). Differences in categorical variables between subgroups were assessed using Pearson’s chi-squared (*χ*^2^) test or Fisher’s exact test, as appropriate. Non-normally distributed continuous variables were compared using the Mann–Whitney *U* test. All statistical tests were two-sided, with a significance threshold set at *α* = 0.05. All descriptive and comparative statistical analyses were performed using IBM SPSS Statistics version 22.0 (IBM Corp., Armonk, NY, USA).

Disproportionality analysis was performed to quantify the statistical association between palivizumab exposure and the reported AEs, and to detect positive signals. This method compares the reporting frequency of a specific AE for palivizumab to the reporting frequency of the same AE for all other drugs in the FAERS database, using a 2 × 2 contingency table (Table 1) (Liu et al. 2019).

**Table 1:** 2 × 2 contingency table of disproportionality analysis

Item	Target adverse events reported	Other adverse events reported	Total
Reports with the target drug	a	b	a + b
All other drugs	c	d	c + d
Total	a + c	b + d	N = a + b + c + d

In this study, we employed four complementary, widely validated disproportionality analysis methods to minimize the risk of false-positive signals and ensure the robustness of our findings: (1) Reporting Odds Ratio (ROR), (2) Proportional Reporting Ratio (PRR), (3) Bayesian Confidence Propagation Neural Network (BCPNN) with Information Component (IC), and (4) Multi-item Gamma Poisson Shrinker (MGPS) with Empirical Bayes Geometric Mean (EBGM). The equations and predefined signal validation criteria for each method are detailed in Table 2.

**Table 2:** Summary of major algorithms used for signal detection

Algorithms	Equation	Criteria
PRR	PRR = (a/(a + c))/(b/(b + d)) χ^2^ = Σ((O − E)2/E); (O = a, E = (a + b)(a + c)/(a + b + c + d)	PRR ≥ 2, χ^2^ ≥ 4, *N* ≥ 3
ROR	ROR = (a/b)/(c/d)	95% CI > 1, *N* ≥ 2
BCPNN	IC = log2a(a + b + c + d)/((a + c)(a + b))	IC025 > 0
MGPS	EBGM = (a(a + b + c + d))/((a + c)(a + b))	EBGM05 > 2

*IC* information component, *IC025* lower 95% credible interval of IC, *EBGM* Empirical Bayes Geometric Mean, *EBGM05* lower 95% confidence bound of EBGM. A signal is positive when IC025 > 0 or EBGM05 > 2

For the ROR method, a positive signal was defined as an AE with ≥ 3 case reports, a lower bound of the 95% confidence interval (CI) for the ROR > 1, and a two-sided *p*-value < 0.05 (Liu et al. 2019). For the PRR method, a positive signal required a PRR ≥ 2, a*χ*^2^ value ≥ 4, and ≥ 3 case reports. For the BCPNN method, a positive signal was defined as an AE with a lower 95% CI of the IC (IC025) > 0. For the MGPS method, a positive signal required a lower 95% CI of the EBGM (EBGM05) > 2.

To be considered a robust, validated positive signal in this study, an AE was required to meet the signal criteria for all four disproportionality analysis methods. A novel signal was defined as a positive signal not listed in the FDA-approved prescribing information for palivizumab. The requirement that a positive signal must meet the criteria of all four methods is intentionally conservative. This approach minimizes false‑positive signals in exploratory FAERS analyses, where multiple testing and confounding are major concerns. The four methods differ in their sensitivity and specificity: ROR and PRR are frequentist measures with high sensitivity but lower specificity, while BCPNN and MGPS are Bayesian shrinkage estimators that stabilize variance for rare events and reduce false positives. Combining them provides a balanced, robust signal detection strategy. Stratified signal detection analyses were also performed for serious vs. non-serious AEs, as defined by the FDA criteria (serious AEs include those resulting in death, life-threatening illness, hospitalization or prolongation of hospitalization, persistent or significant disability, congenital anomaly, or required intervention to prevent permanent impairment).

### Data availability

The FAERS database utilized in this study is available at: https://fis.fda.gov/extensions/FPD-QDE-FAERS/FPD-QDE-FAERS.html.

## Results

### Demographic and clinical characteristics of palivizumab-associated adverse event reports

This 20-year retrospective study (Q1 2004 to Q2 2024) extracted 21,558,936 total adverse event (AE) reports from the FAERS database. After deduplication and filtering, 14,120 eligible reports listing palivizumab as the primary suspected drug were included, accounting for 0.065% of all FAERS reports in the study period (full characteristics detailed in Table 3).

**Table 3:** Clinical characteristics of patients treated with palivizumab

Characteristics	*n* (%)
Sex	
Female (%)	5582 (39.53)
Male (%)	7306 (51.74)
Not specified (%)	1232 (8.73)
Age (years)	
0–27 days (%)	1776 (12.58)
28 days–23 months (%)	6484 (45.92)
2–11 years (%)	213 (1.51)
12–17 years (%)	5 (0.04)
18–44 years (%)	8 (0.06)
45–64 years (%)	1 (0.01)
65–74 years (%)	0 (0.00)
≥ 75 years (%)	1 (0.01)
Not specified (%)	5632 (39.89)
Age (years)	
*N* (missing)	8488 (5632)
Mean (SD)	0.61 (1.72)
Median (Q1, Q3)	0.42 (0.16, 0.91)
Min, max	0.00, 96.00
Report year	
2004 (%)	112 (0.79)
2005 (%)	132 (0.93)
2006 (%)	250 (1.77)
2007 (%)	358 (2.54)
2008 (%)	195 (1.38)
2009 (%)	224 (1.59)
2010 (%)	462 (3.27)
2011 (%)	563 (3.99)
2012 (%)	589 (4.17)
2013 (%)	340 (2.41)
2014 (%)	473 (3.35)
2015 (%)	583 (4.13)
2016 (%)	815 (5.77)
2017 (%)	608 (4.31)
2018 (%)	574 (4.07)
2019 (%)	941 (6.66)
2020 (%)	1931 (13.68)
2021 (%)	1305 (9.24)
2022 (%)	1788 (12.66)
2023 (%)	1226 (8.68)
2024 (%)	651 (4.61)
Reporter	
Consumer (%)	4577 (32.42)
Lawyer (%)	3 (0.02)
Not specified (%)	1580 (11.19)
Other health professional (%)	2662 (18.85)
Pharmacist (%)	2583 (18.29)
Physician (%)	2715 (19.23)
Geographical distribution	
North America (%)	8982 (63.61)
Europe (%)	2814 (19.93)
South America (%)	829 (5.87)
Asia (%)	576 (4.08)
Africa (%)	187 (1.32)
Oceania (%)	11 (0.08)
Reported countries	
USA (%)	8699 (61.61)
Ireland (%)	585 (4.14)
Netherlands (%)	569 (4.03)
Colombia (%)	535 (3.79)
Germany (%)	530 (3.75)
Outcomes	
Life threatening (%)	331 (2.34)
Hospitalization—initial or prolonged (%)	8610 (60.98)
Disability (%)	45 (0.32)
Death (%)	1711 (12.12)
Congenital anomaly (%)	34 (0.24)
Required intervention to prevent (%)	16 (0.11)
Other (%)	2851 (20.19)
Adverse event occurrence time (days)	
0–30 days (%)	2243 (15.89)
31–60 days (%)	1230 (8.71)
61–90 days (%)	749 (5.30)
91–120 days (%)	505 (3.58)
121–150 days (%)	265 (1.88)
151–180 days (%)	155 (1.10)
181–360 days (%)	286 (2.03)
> 360 days (%)	193 (1.37)
Missing or outlier (less than 0) (%)	8494 (60.16)
Adverse event occurrence time (days)	
*N* (missing)	5626 (8494)
Mean (SD)	123.16 (463.04)
Median (Q1, Q3)	43.00 (14.00, 90.00)
Min, max	0.00, 4999.00
Weight (kg)	
*N* (missing)	5526 (8594)
Mean (SD)	7.94 (107.62)
Median (Q1, Q3)	6.01 (4.22, 8.00)
Min, max	0.07, 8000.00

Gender data were available for 91.27% of cases, with a male predominance (51.74% vs. 39.53% female, male-to-female ratio 1.31:1). Among cases with valid age data (60.11% of total), the cohort was overwhelmingly pediatric, consistent with palivizumab’s labeled indication: 45.92% of cases were infants aged 28 days to 23 months, 12.58% were neonates aged 0 to 27 days, and fewer than 2% of cases were children aged 2 years and older. The overall mean age was 0.61 ± 1.72 years (median 0.42 years, IQR 0.16–0.91 years), confirming the predominantly infantile study population.

Temporal analysis showed a marked surge in AE reports starting in 2020, with 1931 reports (13.68% of total) that year, nearly double the 941 reports in 2019, with sustained high reporting through 2022. Most reports were submitted by healthcare professionals (56.37% total, including physicians, pharmacists, and allied health providers), with 32.42% from consumers (predominantly patient caregivers). Geographically, 63.61% of reports originated from North America (61.61% from the USA alone), followed by Europe (19.93%).

The median time from palivizumab administration to AE onset (TTO) was 43 days (IQR 14–90 days), with over 30% of events occurring within the first 90 days of dosing, and only 1.37% of events occurring more than 1 year after the last dose. The most frequent serious outcome was hospitalization (initial or prolonged), reported in 60.98% of cases, followed by death (12.12% of reports); other serious outcomes including life-threatening illness, disability, and congenital anomaly accounted for less than 3% of cases combined.

### System organ class-level safety signal detection

**Fig. 2:**
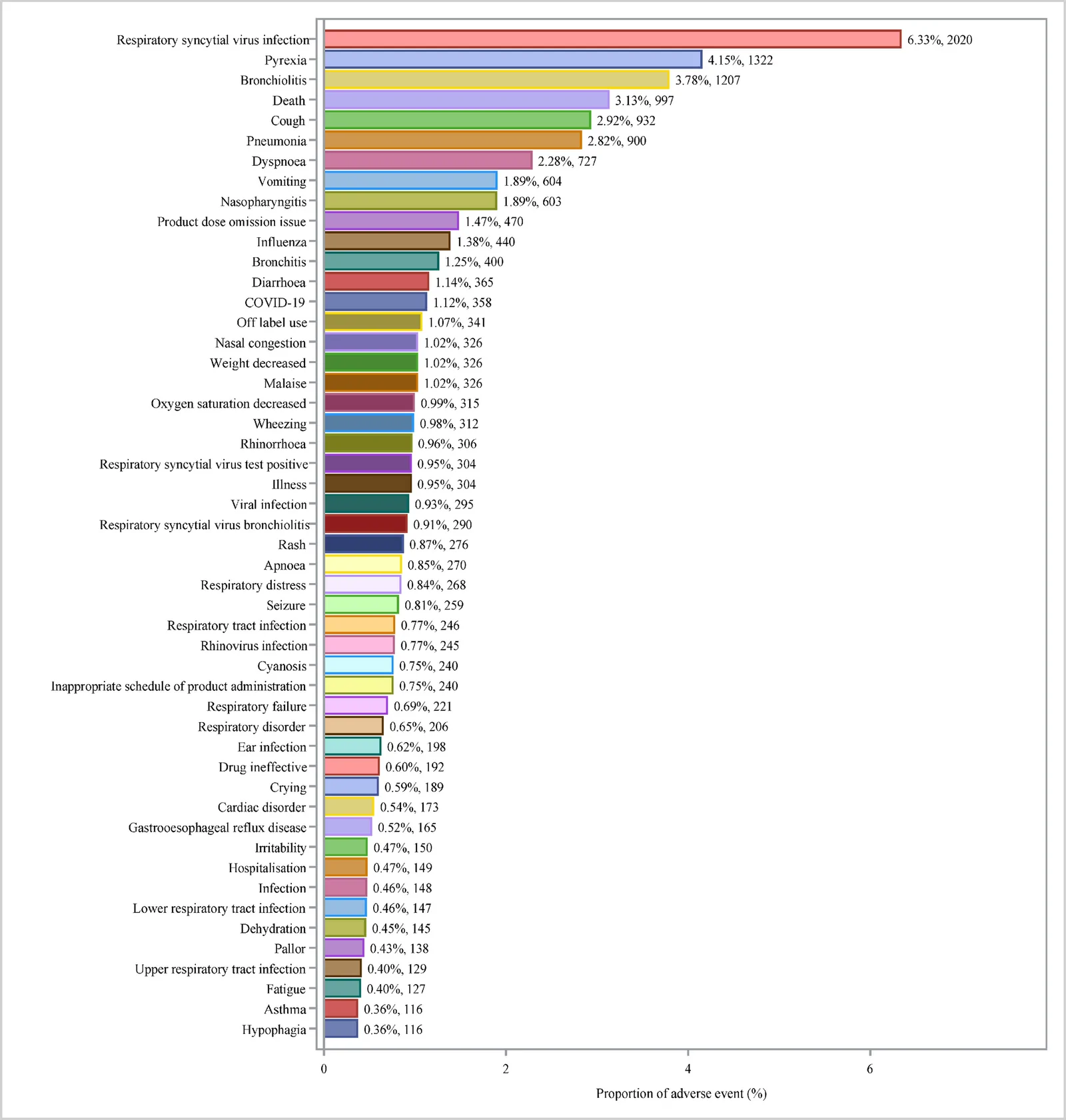
Percentage of signals for palivizumab at the SOC level

Standardized coding with MedDRA version 27.0 identified 27 SOCs across palivizumab-associated AE reports (signal distribution in Fig. 2, full metrics in Table 4). Of these, 3 SOCs met the predefined validation criteria for a positive signal across all four disproportionality analysis methods (ROR, PRR, BCPNN, MGPS).

**Table 4:** Signal strength of AEs at the SOC level in FAERS database

System organ class (SOC)	SOC code	Case reports	ROR(95% CI)	PRR(95% CI)	Chi_Square	IC(IC025)	EBGM(EBGM05)
Infections and infestations*	10021881	9683	7.93 (7.74, 8.12)	5.83 (5.73, 5.92)	40,695.4	2.54 (2.50)	5.81 (5.67)
Respiratory, thoracic and mediastinal disorders ^#^,*	10038738	5672	4.39 (4.26, 4.52)	3.79 (3.70, 3.88)	12,174.0	1.92 (1.88)	3.78 (3.67)
General disorders and administration site conditions^#^	10018065	4955	0.87 (0.84,0.90)	0.89 (0.87, 0.91)	80.39	− 0.17 (− 0.21)	0.89 (0.86)
Gastrointestinal disorders	10017947	1978	0.71 (0.68, 0.74)	0.73 (0.70, 0.76)	218.25	− 0.46 (− 0.52)	0.73 (0.70)
Investigations	10022891	1825	0.92 (0.88, 0.97)	0.93 (0.89, 0.97)	10.54	− 0.11 (− 0.18)	0.93 (0.89)
Injury, poisoning and procedural complications^@^	10022117	1600	0.46 (0.44, 0.49)	0.49 (0.47, 0.51)	946.82	− 1.03 (− 1.10)	0.49 (0.47)
Nervous system disorders^#^	10029205	983	0.34 (0.32, 0.36)	0.36 (0.34, 0.38)	1213.77	− 1.47 (− 1.56)	0.36 (0.34)
Metabolism and nutrition disorders	10027433	837	1.21 (1.13, 1.30)	1.21 (1.13, 1.29)	30.62	0.27 (0.17)	1.21 (1.13)
Skin and subcutaneous tissue disorders^#^	10040785	809	0.46 (0.43, 0.49)	0.47 (0.44, 0.50)	506.58	− 1.08 (− 1.19)	0.47 (0.44)
Cardiac disorders^#^	10007541	804	0.95 (0.88, 1.02)	0.95 (0.89, 1.02)	2.23	− 0.07 (− 0.18)	0.95 (0.89)
Vascular disorders	10047065	536	0.78 (0.72, 0.85)	0.78 (0.72, 0.85)	32.51	− 0.35 (− 0.48)	0.78 (0.72)
Psychiatric disorders	10037175	464	0.25 (0.22, 0.27)	0.26 (0.23, 0.28)	1063.17	− 1.96 (− 2.10)	0.26 (0.23)
Surgical and medical procedures^@^	10042613	436	1.02 (0.93, 1.13)	1.02 (0.93, 1.12)	0.24	0.03 (− 0.11)	1.02 (0.93)
Congenital, familial and genetic disorders*	10010331	277	2.88 (2.56, 3.24)	2.86 (2.54, 3.22)	335.73	1.51 (1.33)	2.86 (2.54)
Blood and lymphatic system disorders^#^	10005329	203	0.37 (0.33, 0.43)	0.38 (0.33, 0.43)	210.75	− 1.40(− 1.60)	0.38 (0.33)
Immune system disorders^#^	10021428	182	0.52 (0.45, 0.60)	0.52 (0.45, 0.60)	81.59	− 0.94 (− 1.15)	0.52 (0.45)
Musculoskeletal and connective tissue disorders	10028395	157	0.09 (0.08, 0.11)	0.09 (0.08, 0.11)	1428.75	− 3.40 (− 3.62)	0.09 (0.08)
Eye disorders	10015919	129	0.20 (0.17, 0.24)	0.20 (0.17, 0.24)	408.41	− 2.29 (− 2.54)	0.20 (0.17)
Renal and urinary disorders	10038359	78	0.13 (0.10, 0.16)	0.13 (0.10, 0.16)	474.62	− 2.97 (− 3.28)	0.13 (0.10)
Hepatobiliary disorders^#^	10019805	71	0.24 (0.19, 0.30)	0.24 (0.19, 0.31)	169.18	− 2.04 (− 2.37)	0.24 (0.19)
Ear and labyrinth disorders^#^	10013993	43	0.31 (0.23, 0.42)	0.31 (0.23, 0.42)	66.47	− 1.69 (− 2.10)	0.31 (0.23)
Neoplasms benign, malignant and unspecified (incl cysts and polyps)^@^	10029104	43	0.05 (0.04, 0.07)	0.05 (0.04, 0.07)	783.55	− 4.30 (− 4.70)	0.05 (0.04)
Product issues^@^	10077536	40	0.08 (0.06, 0.11)	0.08 (0.06, 0.11)	440.67	− 3.67 (− 4.09)	0.08 (0.06)
Social circumstances^@^	10041244	31	0.21 (0.15, 0.30)	0.21 (0.15, 0.30)	92.92	− 2.25 (− 2.73)	0.21 (0.15)
Reproductive system and breast disorders	10038604	27	0.09 (0.06, 0.14)	0.09 (0.06, 0.14)	237.34	− 3.41 (− 3.91)	0.09 (0.06)
Pregnancy, puerperium and perinatal conditions	10036585	16	0.12 (0.07, 0.19)	0.12 (0.07, 0.19)	108.42	− 3.11 (− 3.73)	0.12 (0.07)
Endocrine disorders	10014698	11	0.14 (0.08, 0.25)	0.14 (0.08, 0.25)	60.39	− 2.88 (− 3.60)	0.14 (0.08)

Ranked by case reports

^*^Potential significant SOCs that simultaneously met four criteria

^#^SOCs that were mentioned in the drug leaflet

^@^SOCs that were excluded for further analyses

The strongest positive signal was observed for Infections and infestations (*n* = 9683, ROR = 7.93, 95% CI 7.74–8.12), followed by Respiratory, thoracic and mediastinal disorders (*n* = 5672, ROR = 4.39, 95% CI 4.26–4.52), and Congenital, familial and genetic disorders (*n* = 277, ROR = 2.88, 95% CI 2.56–3.24). Cross-referencing with the FDA-approved prescribing information confirmed Respiratory, thoracic and mediastinal disorders as a known, labeled adverse event category, while the other two SOCs represented novel signals. An additional 8 SOCs aligned with the labeled safety profile of palivizumab, while SOCs including Gastrointestinal disorders, Metabolism and nutrition disorders, and Vascular disorders did not meet all signal criteria but had substantial case volumes warranting further evaluation. SOCs unrelated to the pharmacological effects of palivizumab (e.g., injury, procedural complications, neoplasms) were excluded from further analysis.

### Preferred term-level safety signal detection

At the PT level, 170 individual AEs met the positive signal criteria across all four analysis methods, with the top 30 PTs ranked by the stringent EBGM method presented in Table 5. The strongest positive signals were almost exclusively RSV-related: the top 3 events were respiratory syncytial virus test positive (ROR = 2240.98, 95% CI 1887.14–2661.18), respiratory syncytial virus bronchiolitis (ROR = 1766.55, 95% CI 1497.79–2083.53), and bronchiolitis (ROR = 1108.00, 95% CI 1029.94–1191.98), with 8 of the top 30 PTs directly associated with RSV infection.

**Table 5:** Signal intensity of AEs at the PT level ranked by EBGM

System organ class (SOC)	Preferred term (PT)	Case count	ROR (95% CI)	PRR (95% CI)	Chi_Square	IC (IC025)	EBGM (EBGM05)	Proportion of serious outcomes (%)
Investigations	Respiratory syncytial virus test positive	304	2240.98 (1887.14, 2661.18)	2219.63 (1870.47, 2633.96)	289,651.3427	9.90 (7.65)	954.22 (803.55)	82.89
Infections and infestations	Respiratory syncytial virus bronchiolitis	290	1766.55 (1497.79, 2083.53)	1750.50 (1485.27, 2063.08)	247,721.5981	9.74 (7.55)	855.69 (725.51)	99.31
Infections and infestations	Bronchiolitis	1207	1108.00 (1029.94, 1191.98)	1066.10 (992.68, 1144.96)	784,342.2782	9.35 (8.63)	651.40 (605.51)	98.51
Infections and infestations	Subglottic laryngitis	12	872.69 (434.21, 1753.96)	872.36 (434.12, 1753.00)	6863.428975	9.16 (2.74)	573.61 (285.40)	100.00
Infections and infestations	Respiratory syncytial virus bronchitis	29	836.77 (535.74, 1306.95)	836.01 (535.40, 1305.41)	16,124.28707	9.12 (4.22)	557.67 (357.05)	100.00
Infections and infestations	Respiratory syncytial virus infection	2020	823.57(780.34, 869.20)	771.47 (732.70, 812.29)	1,063,718.285	9.04 (8.63)	528.21 (500.48)	85.74
Respiratory, thoracic and mediastinal disorders	Childhood asthma	8	371.65 (172.74, 799.63)	371.56 (172.72, 799.30)	2418.964461	8.25 (2.08)	304.19 (141.38)	100.00
Gastrointestinal disorders	Infantile spitting up	53	311.45 (232.26, 417.65)	310.94 (231.97, 416.79)	13,806.46509	8.04 (5.07)	262.34 (195.63)	92.45
Infections and infestations	Pneumonia respiratory syncytial viral	79	229.89 (181.67, 290.91)	229.32 (181.31, 290.04)	15,793.01199	7.66 (5.50)	201.78 (159.46)	100.00
Nervous system disorders	Infant irritability^#^	8	222.99 (106.63, 466.35)	222.94 (106.62, 466.16)	1559.586201	7.62 (2.09)	196.83 (94.12)	50.00
Respiratory, thoracic and mediastinal disorders	Bronchopulmonary dysplasia	77	206.16 (162.67, 261.28)	205.66 (162.36, 260.52)	13,964.99786	7.52 (5.43)	183.25 (144.59)	96.10
Nervous system disorders	Fontanelle bulging	11	202.18 (108.14, 378.01)	202.11 (108.12, 377.81)	1963.905004	7.50 (2.62)	180.42 (96.50)	100.00
General disorders and administration site conditions	Sudden infant death syndrome	46	188.32 (138.82, 255.48)	188.05 (138.67, 255.02)	7693.419606	7.40 (4.76)	169.14 (124.68)	100.00
Investigations	Human metapneumovirus test positive	10	187.93 (97.73, 361.35)	187.87 (97.72, 361.17)	1670.984877	7.40 (2.46)	168.99 (87.89)	100.00
Infections and infestations	Rhinovirus infection	245	167.67 (146.97, 191.28)	166.39 (145.98, 189.65)	36,633.00788	7.24 (6.36)	151.42 (132.73)	92.24
Infections and infestations	Rotavirus infection	38	159.03 (114.00, 221.85)	158.84 (113.91, 221.50)	5443.193527	7.18 (4.47)	145.15 (104.05)	100.00
Metabolism and nutrition disorders	Oligodipsia	16	153.83 (92.17, 256.72)	153.75 (92.15, 256.53)	2223.631082	7.14 (3.20)	140.89 (84.42)	100.00
Respiratory, thoracic and mediastinal disorders	Use of accessory respiratory muscles	31	140.99 (97.70, 203.44)	140.85 (97.64, 203.18)	3970.156557	7.02 (4.16)	129.98 (90.08)	96.77
Infections and infestations	Roseola	6	139.36 (60.59, 320.53)	139.34 (60.59, 320.43)	760.6675925	7.01 (1.60)	128.69 (55.95)	66.67
Infections and infestations	Gastroenteritis rotavirus	19	129.74 (81.34, 206.95)	129.67 (81.32, 206.77)	2251.248922	6.91 (3.44)	120.41 (75.49)	100.00
Respiratory, thoracic and mediastinal disorders	Pulmonary vein stenosis	8	128.65 (62.67, 264.09)	128.62 (62.66, 263.98)	940.6452087	6.90 (2.08)	119.50 (58.21)	100.00
Infections and infestations	Croup infectious	43	121.61 (89.22, 165.77)	121.45 (89.13, 165.48)	4788.830321	6.82 (4.54)	113.29 (83.11)	88.37
Metabolism and nutrition disorders	Poor feeding infant	64	119.80 (92.94, 154.42)	119.56 (92.80, 154.04)	7022.578547	6.80 (5.00)	111.65 (86.62)	95.31
Gastrointestinal disorders	Post-tussive vomiting	6	115.33 (50.42, 263.80)	115.31 (50.42, 263.71)	636.0593011	6.75 (1.60)	107.94 (47.19)	100.00
Gastrointestinal disorders	Teething	15	112.52 (66.70, 189.82)	112.47 (66.68, 189.69)	1552.718159	6.72 (3.06)	105.44 (62.50)	60.00
Infections and infestations	Metapneumovirus infection	36	111.80 (79.77, 156.69)	111.68 (79.71, 156.46)	3701.447919	6.71 (4.29)	104.75 (74.74)	94.44
Psychiatric disorders	Breath holding	13	107.12 (61.14, 187.69)	107.08 (61.12, 187.57)	1283.888536	6.65 (2.84)	100.69 (57.47)	100.00
Respiratory, thoracic and mediastinal disorders	Chronic respiratory disease	16	104.15 (62.84, 172.59)	104.09 (62.83, 172.47)	1537.933862	6.62 (3.15)	98.05 (59.17)	93.75
Vascular disorders	Kawasaki’s disease	12	85.78 (48.02, 153.24)	85.74 (48.01, 153.15)	956.0543614	6.35 (2.68)	81.61 (45.68)	100.00
Infections and infestations	Enterovirus infection	31	82.88 (57.78, 118.89)	82.80 (57.74, 118.74)	2386.997427	6.30 (4.00)	78.94 (55.03)	93.55

^#^PTs that were mentioned in the drug label

Notably, only 4 of the top 30 PTs were listed as known adverse events in the palivizumab prescribing information, with the remaining 26 representing novel signals. Key novel signal categories included gastrointestinal events (infantile spitting up—defined as non‑bilious, non‑projectile regurgitation, typically mild; post-tussive vomiting), metabolism and nutrition disorders (oligodipsia, poor infant feeding), rare immune-mediated and vascular conditions (Kawasaki's disease, roseola), non-RSV infectious events (rhinovirus, rotavirus, metapneumovirus infection), and additional respiratory events (subglottic laryngitis, infectious croup, childhood asthma). Detailed clinical characteristics for the 26 novel unlabeled PTs, including case counts, age distribution, sex, and proportion of serious outcomes, are provided in Supplementary Table S1.

## Discussion

This 20-year retrospective pharmacovigilance study is the largest and longest-term real-world analysis of the post-marketing safety profile of palivizumab to date, based on 14,120 palivizumab-associated adverse event (AE) reports from the FAERS database. Using four complementary disproportionality analysis methods, we confirmed the generally favorable safety profile of palivizumab established in pre-approval clinical trials (Giunchi et al. 2023; Johnson et al. 1997), while also identifying multiple novel signals at both the SOC and PT levels, providing critical evidence for clinical practice and post-marketing surveillance.

The demographic profile of the study cohort was fully consistent with the labeled indication of palivizumab recommended by clinical guidelines (Caserta et al. 2023), with the majority of cases concentrated in infants aged 28 days to 23 months. The observed male predominance in AE reports is not indicative of a sex-specific difference in drug safety, but rather reflects the higher rate of palivizumab use in male infants, as male sex is an established independent risk factor for severe RSV infection (Fenton et al. 2004). The near-doubling of AE reports in 2020 was not driven by increased drug utilization, as evidenced by global sales data (Nair et al. 2010), but most likely by the heightened global awareness of respiratory viral infections and adverse drug events during the COVID-19 pandemic, a trend observed for multiple respiratory therapeutics in FAERS (Messina et al. 2022). The majority of reports were submitted by healthcare professionals, supporting the validity and reliability of the dataset, with geographic distribution aligned with the global marketing and clinical use of palivizumab (Mori et al. 2023).

Hospitalization (initial or prolonged) was the most frequent serious outcome, reported in 60.98% of cases. The estimated real-world hospitalization rate was slightly higher than that reported in the landmark pre-approval randomized controlled trial (Johnson et al. 1997), which is attributable to the inclusion of infants with complex comorbidities and severe underlying illness in our real-world cohort—populations typically excluded from randomized controlled trials (Heilman 1990). Death was reported in 12.12% of cases, but these fatal outcomes cannot be directly attributed to palivizumab exposure, as they occurred predominantly in infants with extreme prematurity, congenital heart disease, or immunodeficiency, who have an inherently high baseline mortality risk. This finding is consistent with previous clinical trials that found no significant difference in all-cause mortality between palivizumab and placebo groups (Resch 2017; Rothman et al. 2004).

The strongest positive signals identified were for RSV-related events, which do not indicate that palivizumab increases the risk of RSV infection. Instead, these signals reflect the well-documented ~ 10% breakthrough infection rate of palivizumab prophylaxis (Giunchi et al. 2023), as well as confounding by indication: infants prescribed palivizumab are at high risk of RSV infection and undergo far more frequent RSV testing than the general pediatric population. We also identified two novel SOC-level signals: Infections and infestations and Congenital, familial and genetic disorders. The congenital disorder signal is almost certainly explained by confounding by indication and pre‑existing comorbidities, as infants with congenital disorders are at higher risk for severe RSV infection and thus more likely to receive palivizumab. This signal should not be interpreted as a drug‑induced effect (Caserta et al. 2023). At the PT level, we identified multiple novel signals, including gastrointestinal events (infantile spitting up, post-tussive vomiting), which align with prior descriptive analyses of pediatric AE reports (Shi et al. 2017) and placebo-controlled trials that identified cough and vomiting as common events in palivizumab recipients, as well as metabolic disorders (oligodipsia), and rare immune-mediated conditions (Kawasaki’s disease, roseola). The Kawasaki’s disease signal has a speculative biological hypothesis involving the murine‑derived sequence of palivizumab (Yao et al. 2024); however, this remains strictly hypothesis‑generating. Given the multifactorial etiology of Kawasaki’s disease and the inherent limitations of spontaneous reporting data, this signal should not be interpreted as evidence of causality and requires validation in large controlled studies.

Previous palivizumab safety studies were limited to small clinical trials, single-center cohorts, or short-term post-marketing analyses (Giunchi et al. 2023; Mori et al. 2023). Our large 20-year dataset enabled the detection of rare, low-frequency safety signals that were underpowered in prior research, filling critical gaps in the real-world safety evidence base. The primary strengths of this study include the large sample size, full post-marketing study period, and use of four complementary disproportionality methods to minimize false-positive signals, which is a widely recommended approach for FAERS-based pharmacovigilance research (Lacaze‐Masmonteil et al. 2002; Liu et al. 2019). Limitations are inherent to spontaneous reporting database research, including underreporting of AEs, lack of a control group, inability to establish causal relationships, confounding by indication, and missing data for some clinical variables, which are well-documented limitations of FAERS-based pharmacovigilance studies (Lacaze‐Masmonteil et al. 2002; Messina et al. 2022). Additional limitations include potential duplicate reporting across different sources, medication errors that may be misclassified as AEs that could influence disproportionality calculations. The observed surge in AE reports beginning in 2020 may also reflect changes in palivizumab prescribing patterns during the COVID‑19 pandemic or updates in FDA data submission practices, in addition to heightened awareness of respiratory adverse events. Furthermore, FAERS does not capture the total number of patients exposed to palivizumab; therefore, reporting rates cannot be interpreted as incidence rates.

These findings have meaningful clinical implications: the novel gastrointestinal and metabolic signals should inform caregiver counseling on symptom monitoring for dehydration and poor feeding, while the Kawasaki’s disease signal suggests clinicians should consider immune-mediated reactions in infants presenting with consistent symptoms after palivizumab administration. Our data also highlight the need for ongoing post-marketing safety monitoring of palivizumab, particularly for rare and immune-mediated events, and prospective controlled studies to validate the novel signals identified in this analysis, in line with current pharmacovigilance best practices for pediatric biologics (Giunchi et al. 2023; Lacaze‐Masmonteil et al. 2002).

## Conclusion

This large-scale, 20-year retrospective pharmacovigilance study provides the most comprehensive characterization of the post-marketing safety profile of palivizumab to date, using data from 14,120 palivizumab-associated adverse event reports submitted to the FAERS database. Our findings confirm the known safety profile of palivizumab established in pre-approval clinical trials, while also identifying multiple novel, previously unreported safety signals at both the SOC and PT levels. Novel signals included those related to non-RSV infections, congenital, familial and genetic disorders, gastrointestinal events (infantile spitting up, post-tussive vomiting), metabolic disorders (oligodipsia, poor feeding), and rare immune-mediated conditions (Kawasaki’s disease, roseola). While these findings do not establish causal relationships between palivizumab exposure and the identified adverse events, they provide critical real-world evidence to guide clinical practice, inform patient counseling, and support ongoing post-marketing safety monitoring of palivizumab. Further prospective, controlled clinical and epidemiological studies are needed to validate these novel signals, investigate potential underlying biological mechanisms, and further define the risk–benefit profile of palivizumab in high-risk pediatric populations. 

## Supplementary Information

Below is the link to the electronic supplementary material.

ESM 1 Supplementary Material 1 (DOC 46.5 KB)

## Data Availability

The FAERS database utilized in this study is available at: https://fis.fda.gov/extensions/FPD-QDE-FAERS/FPD-QDE-FAERS.html.
