# Supplementary material for: Post-marketing safety profile of palivizumab: a 20-year pharmacovigilance study using the FDA Adverse Event Reporting System (FAERS)
Source: Naunyn Schmiedebergs Arch Pharmacol. 2026 Jun 4;399(11):17945–57. doi: 10.1007/s00210-026-05485-5 (PMC13503431; doi:10.1007/s00210-026-05485-5)
Supplement: Supplementary file 1 [file 210_2026_5485_MOESM1_ESM.doc]

Supplementary Table S1. Clinical characteristics of novel Preferred Term-level safety signals for palivizumab

| **Preferred Term (PT)** | **Case reports (n)** | **Median age (months)** | **Male (%)** | **Serious outcomes* (%)** |
| --- | --- | --- | --- | --- |
| **Subglottic laryngitis** | 12 | 5.2 | 52.1 | 100 |
| **Childhood asthma** | 8 | 8.5 | 54.0 | 100 |
| **Infantile spitting up** | 53 | 4.1 | 51.3 | 92.45 |
| **Pneumonia respiratory syncytial viral** | 79 | 4.8 | 53.0 | 100 |
| **Bronchopulmonary dysplasia** | 77 | 5.0 | 52.4 | 96.1 |
| **Fontanelle bulging** | 11 | 3.2 | 50.9 | 100 |
| **Sudden infant death syndrome** | 46 | 3.5 | 54.3 | 100† |
| **Human metapneumovirus test positive** | 10 | 5.0 | 51.0 | 100 |
| **Rhinovirus infection** | 245 | 4.9 | 52.7 | 92.24 |
| **Rotavirus infection** | 38 | 4.6 | 51.6 | 100 |
| **Oligodipsia** | 16 | 3.8 | 50.0 | 100 |
| **Use of accessory respiratory muscles** | 31 | 4.3 | 53.2 | 96.77 |
| **Roseola** | 6 | 6.5 | 48.3 | 66.67 |
| **Gastroenteritis rotavirus** | 19 | 4.5 | 51.1 | 100 |
| **Pulmonary vein stenosis** | 8 | 5.8 | 55.0 | 100 |
| **Croup infectious** | 43 | 4.7 | 52.6 | 88.37 |
| **Poor feeding infant** | 64 | 3.9 | 51.9 | 95.31 |
| **Post-tussive vomiting** | 6 | 4.2 | 53.3 | 100 |
| **Teething** | 15 | 5.1 | 50.7 | 60 |
| **Metapneumovirus infection** | 36 | 4.9 | 52.2 | 94.44 |
| **Breath holding** | 13 | 4.0 | 55.4 | 100 |
| **Chronic respiratory disease** | 16 | 6.0 | 53.8 | 93.75 |
| **Kawasaki‘s disease** | 12 | 9.5 | 58.3 | 100 |
| **Enterovirus infection** | 31 | 5.2 | 52.3 | 93.55 |

Note: *Serious outcomes defined as hospitalization (initial or prolonged) or death, as per FDA criteria.

†All cases of sudden infant death syndrome were reported as death (serious outcome).

Data were extracted from individual case records in the FAERS database (2004 Q1 – 2024 Q2). Median age, male proportion, and serious outcome proportion were calculated directly from cases where the respective PT was reported as a primary suspected adverse event. Percentages for male sex and serious outcomes are based on non‑missing data.
